# Mushroom for Improvement Case Report: The Importance of Involving Mycologists

**DOI:** 10.21980/J8ZW7W

**Published:** 2022-10-15

**Authors:** Gary Bhagat, Marit Tweet, Steven Aks

**Affiliations:** * University of Illinois College of Medicine in Peoria, Department of Emergency Medicine, Peoria, IL; ^ Cook County Health, Department of Emergency Medicine, Chicago, IL; † The Toxicon Consortium, Chicago, IL

## Abstract

A case of acute cholecystitis following giant puffball mushroom ingestion is described. A 64-year-old male ingested what was presumed to be a puffball mushroom, developed epigastric pain several hours later, and went to the emergency department. Patient blood work yielded elevated liver enzymes and bilirubin levels, with no change in coagulation studies. A mycologist associated with our regional poison center confirmed the mushroom to be a giant puffball mushroom which is recognized as a non-poisonous mushroom. After identification of the mushroom and recognition that it was unlikely the cause of the elevated liver enzymes, the workup was expanded, and the patient was found to have acute cholecystitis on imaging. This case highlights the added value of incorporating mycologists when the mushroom in question is available.

**Topics:**

Toxicology, mycology, poison control/center, puffball mushroom, Calvatia, Lycoperdon.

**Figure:**
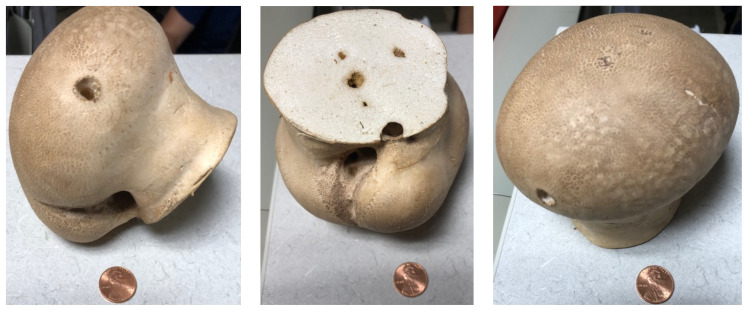


## Brief introduction

Foraging for mushrooms is a popular activity across the world including within the United States. This popularity has led to numerous mycotoxin exposures.^1^ Annually, calls to poison control centers regarding mushroom ingestions typically number in the thousands, with two deaths noted in 2018 from the most recent available data report from the American Association of Poison Control Center, and most of these ingestions never having the mushroom in question identified.^1^–^3^

We present the case of an ingestion of a mushroom that was eventually identified as nontoxic by a local mycologist. This identification allowed for expansion of the workup of the patient’s symptoms, leading to a diagnosis of acute cholecystitis

## Presenting concerns and clinical findings

A 64-year-old Caucasian male, with a past medical history that includes inguinal hernia repair and coronary artery disease, presented to the emergency department (ED) late at night for evaluation of abdominal pain. Earlier in the day, the patient happened upon what he believed to have been a puffball mushroom. The patient had prepared and eaten foraged puffball mushrooms before, so he took the mushroom home, washed it, and opened it to see that it was white on the inside without any obvious yellowing/browning. He then cooked and ate a portion of it. Roughly 6 hours after the initial ingestion the patient began having worsening epigastric abdominal pain. Upon arrival to the ED, patient was noted to have normal vital signs with epigastric abdominal tenderness and no other acute findings on ED physical exam.

## Significant findings

The mushroom displayed here is large and lacks any gills. Small puffball mushrooms can resemble young immature button top *Amanita* type mushrooms. Opening the *Amanita* mushroom should reveal apparent gills and quickly differentiate the two-the puffball mushroom should have a white interior without gills.^4^–^6^

## Patient course

Given the mushroom ingestion, our regional poison control center was contacted by the emergency department, and after reviewing the case, further laboratory testing was recommended, and the photos were sent to local mycologist for identification (Figures 1A–C). The patient symptoms began improving, and he was symptom free. However, his bilirubin and liver enzymes continued to rise with a total bilirubin of 4.2, direct bilirubin 3.0, AST 343, ALT 347, and INR 1.0. Mycologists identified the mushroom as a puffball. Once the mushroom had been identified as a mushroom that is not known to cause any hepatoxicity, our regional poison control center recommended an expansion of the initial work up. The physician ordered an ultrasound that revealed cholelithiasis, acute cholecystitis, hepatic stenosis, and hepatic cysts. After these findings, the surgeons performed a laparoscopic cholecystectomy. The patient recovered from the procedure uneventfully and was discharged from the hospital.

## Discussion

The timing and type of symptoms present in patients after a mushroom ingestion are very important. Traditional teaching states that symptoms presenting early on, within 2–3 hours of ingestion, suggests a gastrointestinal irritant mushroom and are relatively harmless .^1^–^3^

Delayed presentations are those that begin six or more hours after initial ingestion. Symptoms can be mild initially, but the morbidity and mortality is thought to be higher with these presentations. These presentations are most associated with mushrooms from the *Amanita*, *Gyromita*, and *Cortinarius* genera; however, many more identified genera exist.^2^–^5^

Given that severe sequelae are possible, swift identification of the ingested mushroom is key in management of these patients. Certain toxic mushrooms will have obvious features and can be readily identifiable; however, most species fall beyond the scope of the ED provider. Enlisting the help of a mycologist is critical.^3^,^5^

Knowledge of imitators can be of helpful knowledge to the ED provider. Small puffball mushrooms can resemble young immature button top *Amanita* type mushrooms. Opening the *Amanita* mushroom should reveal apparent gills and quickly differentiate the two-the puffball mushroom should have a white interior without gills.^6^–^8^ If the puffball is yellow or brown on the inside, it should not be ingested. It may cause illness, or contain xenobiotics causing adverse effects, such as GI upset.^6^–^8^ In the case described here, the size of the mushroom was more consistent with a puffball. The mushroom was white on the inside at the time of ingestion, and no obvious gills were noted, thus making *Amanita* ingestion less likely.^1^,^5^ It should also be noted that puffball is a broad generalizing term and encompasses multiple genera, including *Calvatia*, *Lycoperdon*, and *Scleroderma*.^9^,^10^

The giant puffball mushroom is widely known to be edible and considered to be a delicacy. It is very possible that any elaborate, fatty meal the patient ingested that evening might have led to his visit for acute cholecystitis. This case highlights the importance of enlisting mycological expertise in identification of the ingested specimen where possible. Once this mushroom was identified, the poison center and the managing service further expanded the workup to etiologies beyond the mushroom and make the diagnosis of acute cholecystitis.

Mushroom ingestions are an infrequent encounter in the emergency department but can have potentially lethal consequences. Key historical features involve the timing of ingestion and the exact symptoms present. Concern for a hepatotoxic mushroom ingestion may distract a clinician from other diagnoses. Having the mushroom present and utilizing poison control and expert mycologists as resources can aid in the management and disposition of the patient. For cases with delayed or atypical presentations, admit the patient for observation, continue supportive care, and continue probing for other possible etiologies causing the patient’s symptoms.

## Supplementary Information






