# Supplementary figures and images for: Mushroom for Improvement Case Report: The Importance of Involving Mycologists
Source: J Educ Teach Emerg Med. 2022 Oct 15;7(4):V1–3. doi: 10.21980/J8ZW7W (PMC10332662; doi:10.21980/J8ZW7W)

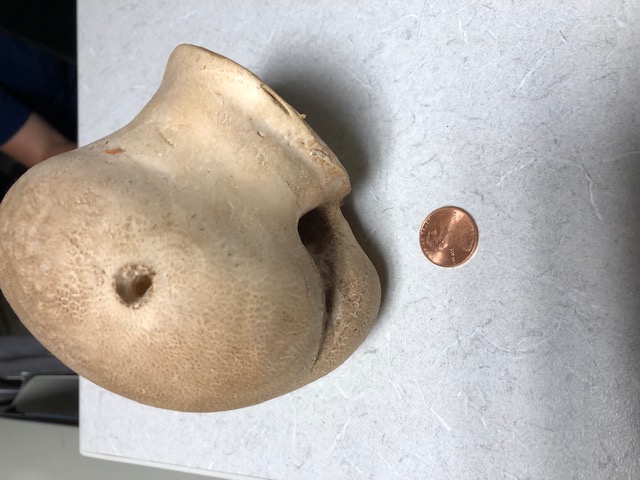

Supplement: Supplementary file 1 [file JETem-7-4-V1-supp1.jpg]

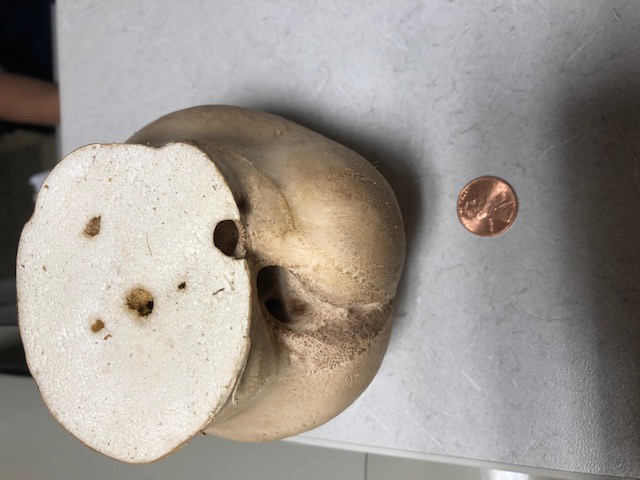

Supplement: Supplementary file 2 [file JETem-7-4-V1-supp2.jpg]

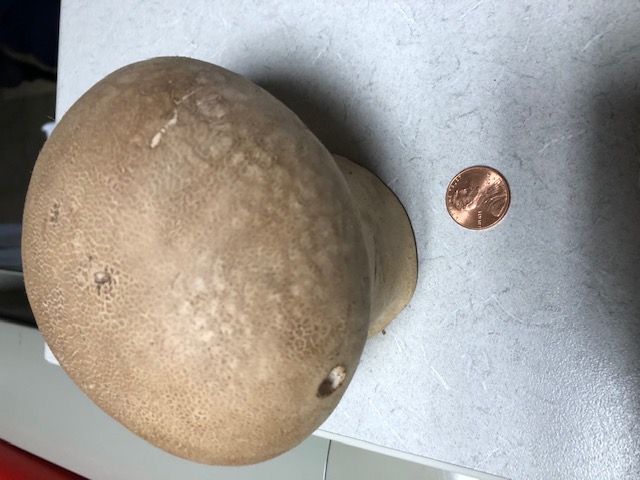

Supplement: Supplementary file 3 [file JETem-7-4-V1-supp3.jpg]
